# Comparative Genomics Analysis and Outer Membrane Vesicle-Mediated Horizontal Antibiotic-Resistance Gene Transfer in Avibacterium paragallinarum

**DOI:** 10.1128/spectrum.01379-22

**Published:** 2022-08-24

**Authors:** Jie Xu, Chen Mei, Yan Zhi, Zhi-xuan Liang, Xue Zhang, Hong-jun Wang

**Affiliations:** a Institute of Animal Husbandry and Veterinary Medicine, Beijing Municipal Academy of Agriculture and Forestry, Beijing, China; Yeungnam University

**Keywords:** *A. paragallinarum*, outer membrane vesicles, whole genome, antibiotic resistance gene, horizontal gene transfer

## Abstract

Avibacterium paragallinarum is the etiological agent of infectious coryza, an acute respiratory disease of chickens that is globally distributed and causes serious economic losses for chicken production. *A. paragallinarum* is a Gram-negative bacterium that releases outer membrane vesicles (OMVs). In this study, a comparative genomic analysis of *A. paragallinarum* isolate P4chr1 and its OMVs was carried out, and the ability to transfer antibiotic resistance genes (ARGs) via the OMVs was studied. Sequencing and data analyses demonstrated that the genomic size of *A. paragallinarum* P4chr1 was approximately 2.77 Mb with a 25 kb tolerance island that covered six types of antibiotics and 11 ARGs. The genomic size of its OMVs was approximately 2.69 Mb, covering 97% of the genomic length and almost all the gene sequences of P4chr1. Purified and DNase-treated *A. paragallinarum* P4chr1 OMVs were cocultured with the antibiotic-sensitive *A. paragallinarum* Modesto strain on an antibiotic (chloramphenicol, erythromycin, tetracycline, or streptomycin)-containing plate, and the corresponding ARGs were detected in the colonies grown on the plates. However, using an antimicrobial susceptibility test, we found that ARGs delivered by OMVs were not persistent but only appeared transiently on the antibiotic-containing plates. Antibiotic resistance and ARGs were lost by the second bacterial passage.

**IMPORTANCE** The functions and roles of OMVs on ARG and virulent gene transfer and dissemination have been reported in numerous Gram-negative bacteria. However, the role of OMVs in mediating antibiotic resistance in *A. paragallinarum* has not been reported. This study is the first report to compare the genomic characteristics of OMVs with its parent *A. paragallinarum* strain and to study *A. paragallinarum* ARG transfer via OMVs. This work has provided useful data for further studies focusing on nonplasmid ARG transfer mediated by *A. paragallinarum* OMVs.

## INTRODUCTION

Infectious coryza is an acute upper respiratory disease of chickens caused by Avibacterium paragallinarum, a Gram-negative bacterium of the genus *Avibacterium* within the family *Pasteurellaceae*. The disease occurs worldwide and leads to serious economic losses in the chicken industry due to the retarded growth of broilers and reduced egg production in layers (1, 2). Disease prevention has involved the use of inactivated multivalent vaccines (based on local prevalent *A. paragallinarum* serotypes), and selected antibiotics have only been administered to diseased flocks (1, 3, 4). Several studies have reported increased infectious coryza outbreaks and have examined antibiotic resistance profiles (3–5), as well as detected antibiotic resistance genes (ARGs) in *A. paragallinarum* (3, 5).

The increase in multidrug-resistant bacterial pathogens is a global threat to both public (6) and animal health (7, 8). However, the rate of development of drug-resistant bacteria is currently surpassing the rate of development of new antibiotics (9). Furthermore, it is becoming evident that bacterial drug resistance can be transferred from food-producing animals to humans through accumulated drug residue in meat and egg products (7, 8). Thus, a better understanding of the mechanisms associated with drug resistance is critical.

Infections caused by Gram-negative bacteria are more difficult to treat than those caused by Gram-positive bacteria, due to the two layers of complex cell membranes that make up the Gram-negative cell wall (10, 11). In order to adapt to adverse environmental conditions, Gram-negative bacteria have evolved globular bi-layered vesicles (diameter, 50 to 500 nm), known as outer membrane vesicles (OMVs), which are produced through the blebbing and pinching-off the bacterial outer membrane without destroying it (6, 12). OMVs contain many components found in the outer membrane of the bacterial cell, such as lipopolysaccharides, phospholipids, membrane proteins, and peptidoglycan components (6, 12). The lumen of the vesicles contains periplasmic proteins, cytosolic components, and nucleic acids (13). OMVs also carry DNA and RNA on their surface, which can be removed by treating OMVs with DNase and RNase, whereas luminal DNA and RNA are not affected by this treatment (14).

Recent studies have revealed a novel mechanism by which antibiotic-susceptible bacteria obtain ARGs from ARG donor bacteria (in the same or different species) using OMVs as vehicles, rather than the three traditional routes, namely, natural transformation, transduction, or conjugation by bacterial cells (6). Although these known mechanisms contribute to the gene flow within bacteria, they have restrictions such as limited genetic load, host specificity, and the type of genetic material that is transferred (6).

To date, the functions and roles of OMVs on ARG and virulent gene transfer and dissemination have been reported in numerous Gram-negative bacteria, such as Acinetobacter baumannii, Escherichia coli, Porphyromonas gingivalis, and Pseudomonas aeruginosa (15–19). However, the role of OMVs in mediating antibiotic resistance in *A. paragallinarum* has not been reported.

Recently, we sequenced the whole genome of a newly isolated *A. paragallinarum* strain, P4chr1 (GenBank accession number CP081939), and identified several ARGs in it. In the current study, we aimed at performing comparative genomics analysis between a multidrug-resistant strain of *A. paragallinarum* P4chr1 and its OMVs, based on genomic sequencing data. In addition, we demonstrated that *A. paragallinarum* P4chr1 OMVs mediated the transfer of aminoglycoside antibiotic genes to a drug-susceptible strain by horizontal gene transfer (HGT).

## RESULTS

### Ultrastructure of OMVs.

**FIG 1:**
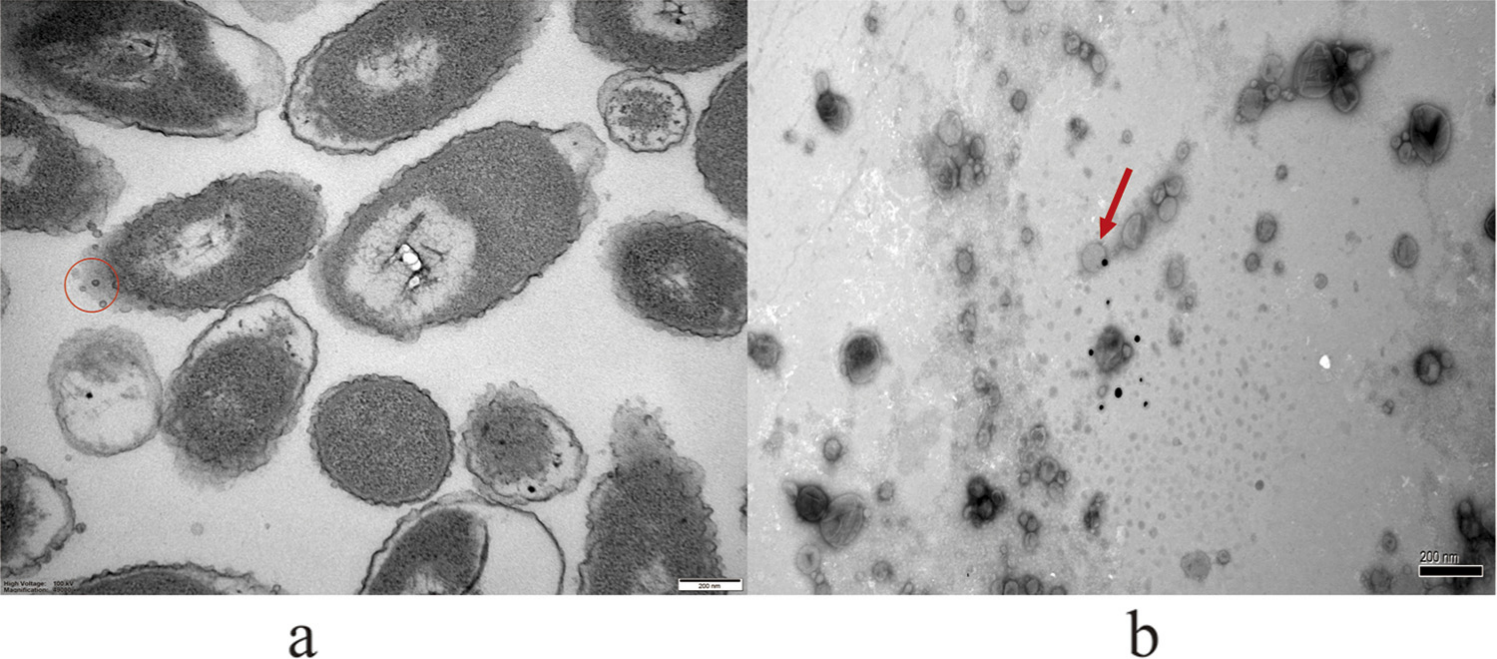
(a) Representative image of *A. paragallinarum* P4chr1 OMVs visualized by transmission electron microscopy. (b) Representative image showing release of OMVs by *A. paragallinarum* strain P4chr1. Vesicles were purified from broth culture by ultracentrifugation and filtered through a 0.45-μm filter. The average diameter of the vesicles was 40 nm. The OMVs were free of bacterial contamination. The red circle and arrow indicate OMVs.

The P4chr1 bacterial solution and extracted OMVs were observed by transmission electron microscopy (TEM). Spherical structures were observed around the *A. paragallinarum* P4chr1 isolates (Fig. 1a), and extracted OMVs showed a similar spherical structure (Fig. 1b). Thus, our findings demonstrated that P4chr1 can secrete OMVs into the environment during growth. The particle size of the OMVs was between 30 and 100 nm, with an average particle size of 40 nm.

### Genomic characterization of *A. paragallinarum* P4chr1.

**FIG 2:**
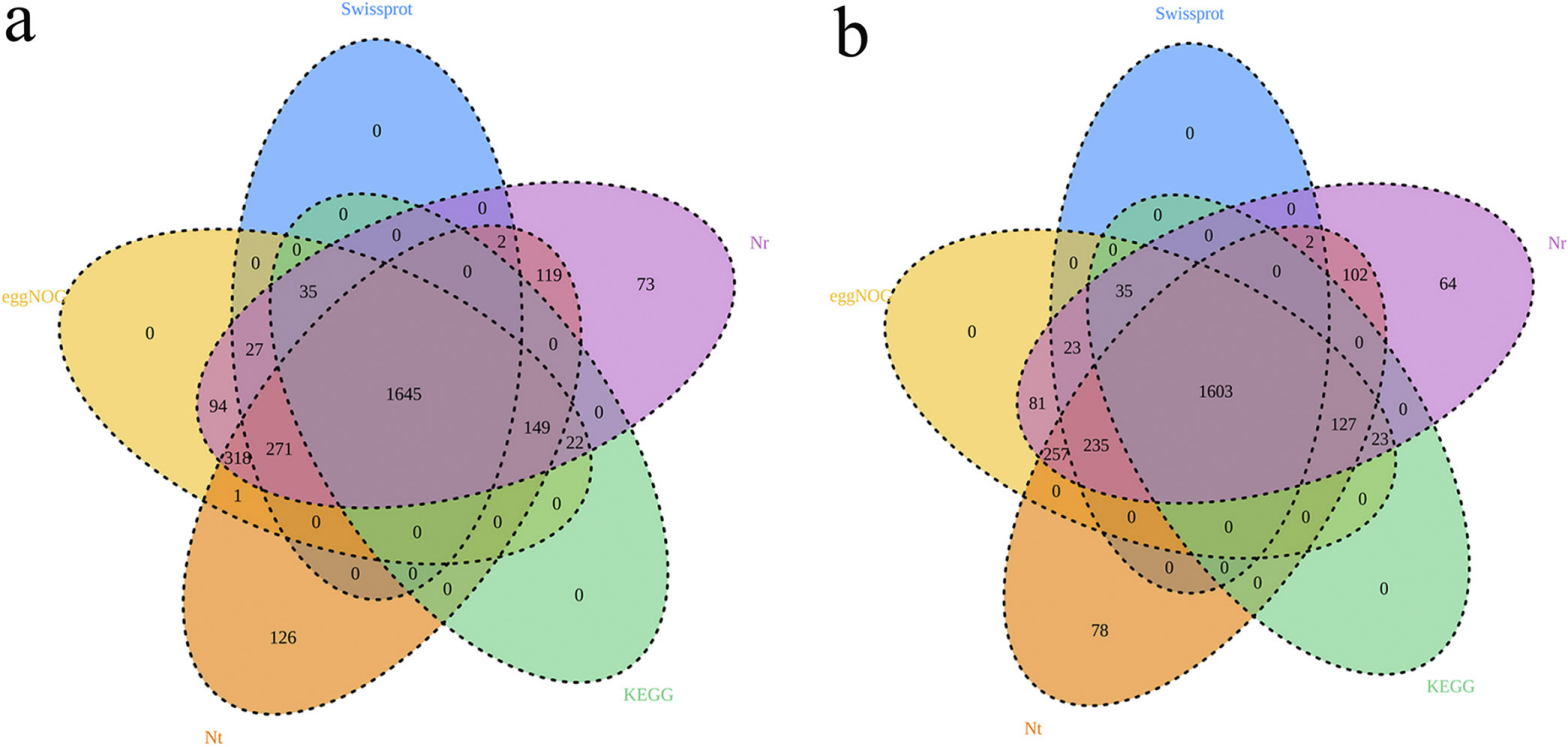
(a) Venn diagram showing the *A. paragallinarum* P4chr1 genome annotated in each database. (b) Venn diagram showing the OMV genome annotated in each database. Different colors represent different databases. Different colored circles have overlapping areas that can be annotated by different databases.

The assembled whole-genome sequence revealed that *A. paragallinarum* P4chr1 harbored a circular chromosomal DNA (2,774,989 bp) with a 41.01% GC content, and it did not carry a plasmid. In total, 2,778 protein-encoding genes were predicted, with a coding percentage of 95.92%. The average gene length was 852 bp. The software ARAGORN was used to predict tRNAs, and the predicted number was 59. The software RNAmmer was used to predict rRNAs, and the predicted number was 15. In addition, the genes were searched against the KEGG, EggNOG, nonredundant (Nr), nucleotide (Nt) and Swiss-Prot databases to annotate the gene description. Among the 2,778 genes predicted in *A. paragallinarum* P4chr1, 2,778 genes were annotated into the COG database, accounting for 92.22% of the predicted genes, which could be divided into 21 categories. In addition, 1,851 genes were annotated into the KEGG database, accounting for 66.63% of the predicted genes. These genes were divided into 38 metabolic pathway types (Fig. 2a) and are described in detail in Table 1.

**TABLE 1:** The genomic status of *A. paragallinarum* P4chr1 annotated in five databases^*a*^

Database	Hit gene no.	Total gene no.	Total perc (%)	Total anno	Total anno (%)
Swiss-Prot	1,980	2,778	71.27	2,896	68.37
EggNOG	2,562	2,778	92.22	2,896	88.47
Nt	2,631	2,778	94.71	2,896	90.85
KEGG	1,851	2,778	66.63	2,896	63.92
Nr	2,755	2,778	99.17	2,896	95.13

a Perc, percentage; anno, annotation.

### Antibiotic resistance of *A. paragallinarum* P4chr1 (serovar A).

**FIG 3:**
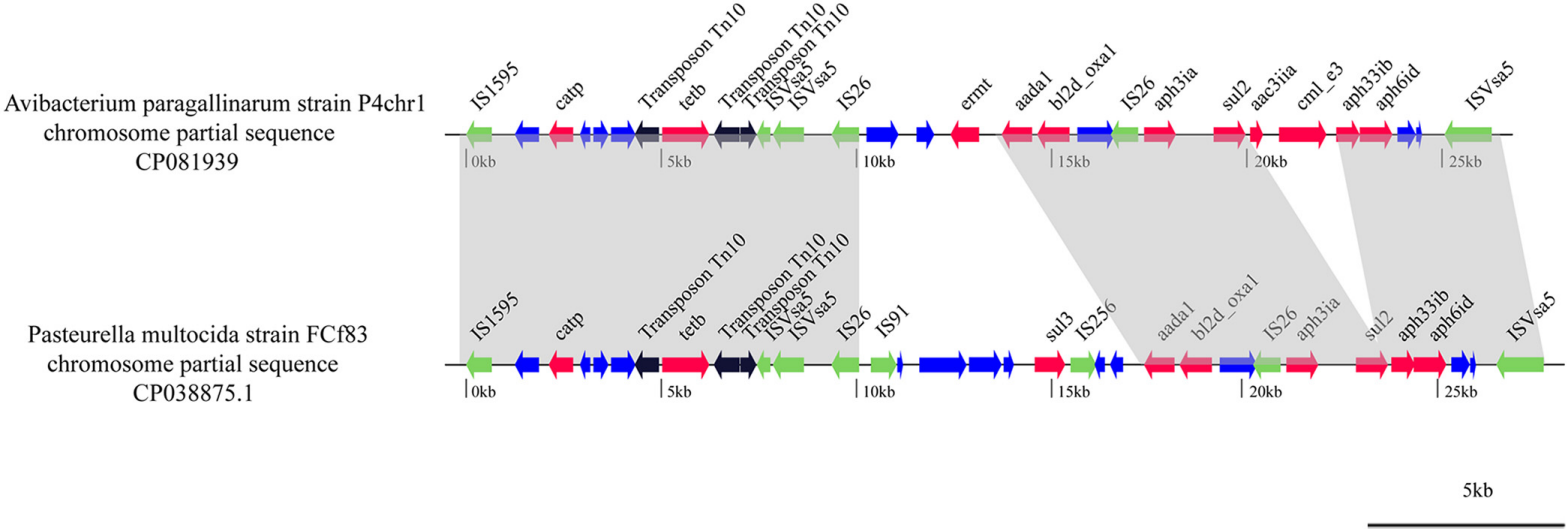
Comparison of *A. paragallinarum* P4chr1 with the corresponding chromosomal regions of the Pasteurella multocida strain FCf83 from China. The arrows indicate the extents and directions of transcription of the genes. ORFs with different functions are presented in various colors. Regions with >99% homology between *A. paragallinarum* P4chr1 and the chromosome of Pasteurella multocida strain FCf83 are indicated by gray shading.

The ResFinder data demonstrated that the chromosomal DNA of *A. paragallinarum* P4chr1 contained 11 ARGs corresponding to 6 categories of antibiotics (the aminoglycoside resistance genes *aph6id*, *aph3ia*, *aac3iia*, *ant2ia*, and *aph33ib*, the beta-lactam resistance gene *bl2d_oxa1,* the MLS [macrolide, lincosamide, and streptogramin B] resistance gene *ermT* the phenicol resistance genes *catP* and *cmL_e3*, the sulfonamide resistance gene *sul2*, and the tetracycline resistance gene *tetB*). The ARGs were all concentrated in a 25-kb fragment of the genome. Sequence comparison analysis revealed that the resistance region of *A. paragallinarum* P4chr1 exhibited high homology to the corresponding region in the chromosomal DNA of Pasteurella multocida strain FCf83 (accession number CP038875) from China (Fig. 3).

Antimicrobial susceptibility testing showed that *A. paragallinarum* P4chr1 was resistant to chloramphenicol, erythromycin, gentamicin, tetracycline, streptomycin, and ampicillin, whereas the recipient strain *A. paragallinarum* Modesto was sensitive to these antibiotics.

### Collinearity analysis of the two genomes.

**FIG 4:**
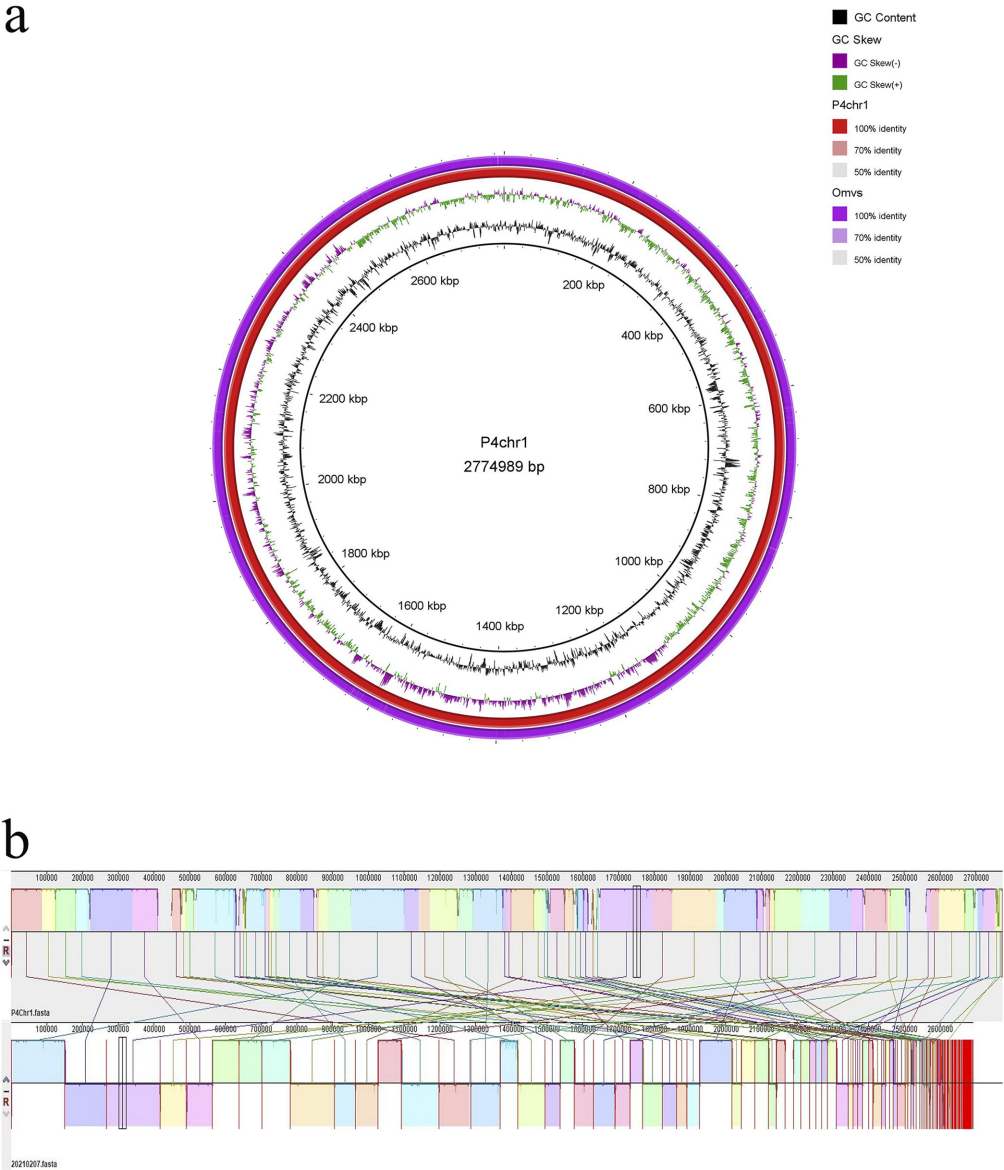
(a) Comparative genomic circle diagram of *A. paragallinarum* P4chr1 and OMVs. The circles show (from outside to inside) OMV genomic sequences, P4chr1 genomic sequences, GC skew, GC content, and scale in kb. (b) Genomic collinearity analysis of *A. paragallinarum* P4chr1 and OMVs. The two areas connected by a line have similar sequences.

The genomic sequence of the OMVs of *A. paragallinarum* strain P4chr1 were composed of 162 contigs for 2,691,804 bp with a 40.92% GC content. The base pair numbers in the OMVs were 97.00% of that found in *A. paragallinarum* P4chr1. The largest contig was 149,543 bp, and the smallest contig was 261 bp. In total, 2,568 protein-encoding genes were predicted. The average gene length was 859 bp. Fig. 2b shows the genomes of the OMVs annotated in various databases. Comparative genomic circle graphs of *A. paragallinarum* P4chr1 and its OMVs indicated that the similarity of the two genomes was greater than 90% (Fig. 4a).

**FIG 5:**
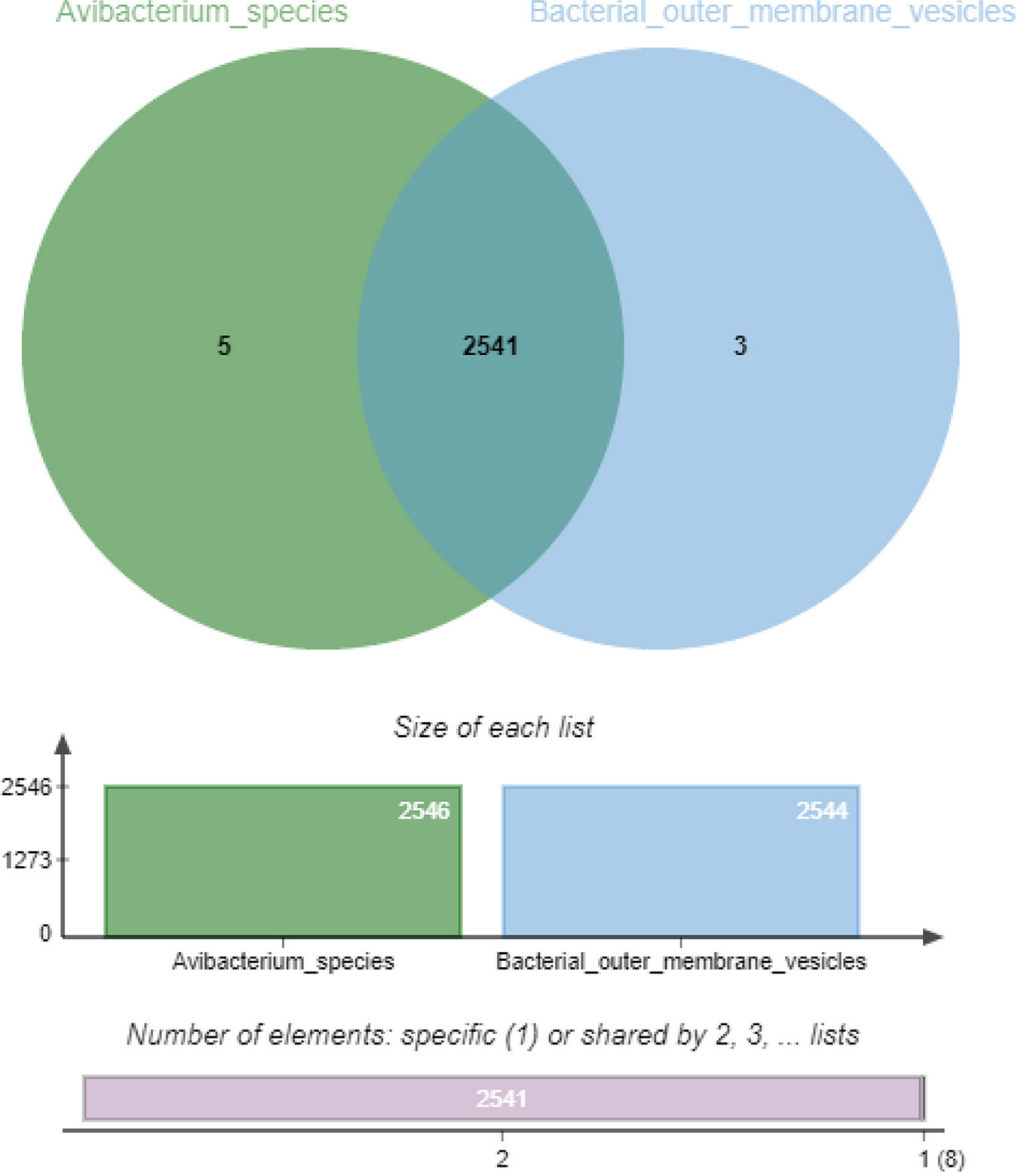
Orthologous cluster analysis of *A. paragallinarum* P4chr1 (*Avibacterium*_species) and OMVs (Bacterial_outer_membrane_vesicle).

The collinearity results indicated that most of the genomic segments of the OMVs had counterparts in the *A. paragallinarum* P4chr1 genome (Fig. 4b). Orthologous cluster analysis of *A. paragallinarum* P4chr1 and its OMVs showed that P4chr1 had 2,546 clusters, while the OMVs had 2,544 homologous clusters, 2,541 of which were shared by P4chr1 and OMVs (Fig. 5). These results indicated that the genome of the OMVs was derived from *A. paragallinarum* P4chr1. Furthermore, our data demonstrated that the OMVs had almost complete genomic sequences of *A. paragallinarum* P4chr1, including some virulence genes and ARGs.

### Transfer of ARGs.

**FIG 6:**
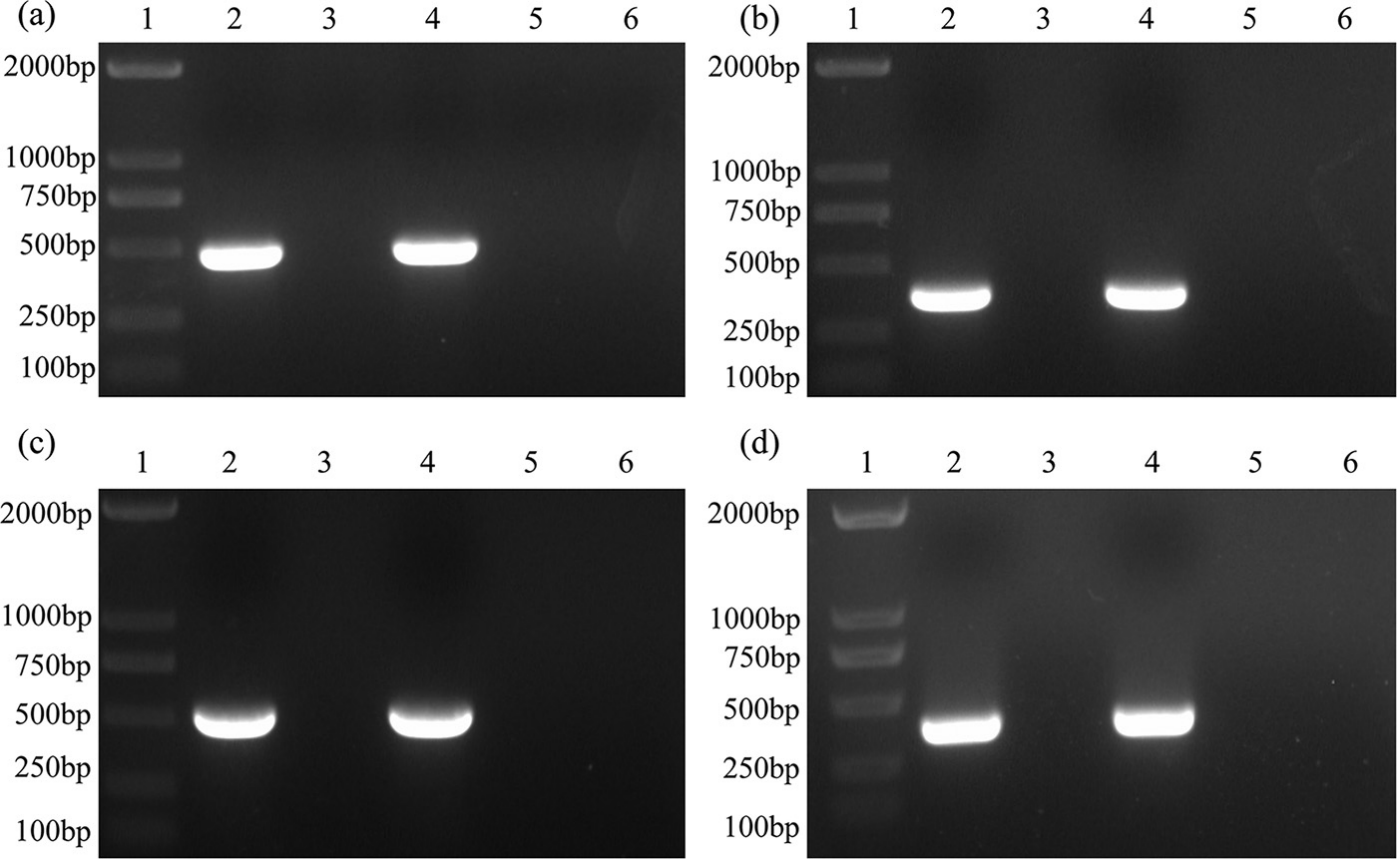
PCR verification of antibiotic resistance genes. (a) *bl2d_oxa1*; (b) *aph33ib*; (c) *cml_e3*; (d) *tetB*. Lane 1, marker 2,000 bp; lane 2, P4chr1; lane 3, Modesto; lane 4, Modesto plus OMVs (single colony from TSA plate with antibiotic); lane 5, Modesto plus OMVs (single colony from TSA plate without antibiotic); lane 6, double-distilled water (ddH_2_O).

First, purified OMVs isolated from *A. paragallinarum* P4chr1 were inoculated with tryptic soy agar (TSA) and broth (TSB) containing supplements. No bacterial growth was detected after 24 or 48 h of incubation, indicating that the OMVs were free of bacterial contamination. Next, *A. paragallinarum* Modesto was transformed with the purified OMVs (Table 2). The transformed colonies produced from the resistant plates were tested for ARGs by PCR. A representative agarose gel showing the corresponding ARG bands is shown in Fig. 6. The sequencing results of the PCR products were also consistent with the ARG sequences annotated at NCBI.

**TABLE 2:** Transformed colony yield obtained on TSA plates under different treatments and their ARG detection by PCR

Group	Treatments	Antibiotic used in TSA plates	CFU/0.1 mL of cells	Transformation frequency^*d*^	PCR^*e*^
Control A^*a*^	Modesto + OMV	/	4.76 × 10^7^	NA	_
Control B^*b*^	Modesto + OMV + DNase	/	3.54 × 10^7^	NA	_
Control C^*c*^	Modesto	Cultured on 4 different antibiotic plates	0	NA	/
Test 1^tet^	Modesto + OMV + DNase	Tetracycline (16 μg/mL)	13	2.73 × 10^−7^	+
Test 2^amp^	Modesto + OMV + DNase	Ampicillin (64 μg/mL)	16	4.52 × 10^−7^	+
Test 3^chl^	Modesto + OMV + DNase	Chloramphenicol (16 μg/mL)	9	1.74 × 10^−7^	+
Test 4^str^	Modesto + OMV + DNase	Streptomycin (256 μg/mL)	28	5.42 × 10^−7^	+
P4chr1	NA	NA	NA	NA	+
Modesto	NA	NA	NA	NA	_
H_2_O	NA	NA	NA	NA	_

a Control A was used to determine the number of receptor cells.

b Control B was used to assess the effect of DNase on recipient cells.

c Control C served as a negative control.

d The data were expressed as the mean over three repeated experiments.

e The monoclonal strains on the plate were tested for antibiotic resistance genes using PCR. +, positive; –, negative; /, none; NA, not available.

Antimicrobial susceptibility testing was performed using the donor strain P4chr1, susceptible strain Modesto, and four ARG-transformed Modesto strains. The MIC values in these four strains did not increase compared with the antibiotic-sensitive Modesto strain (Table 3).

**TABLE 3:** Antibiotic susceptibility profiles of *A. paragallinarum* P4chr1, Modesto, and OMV-transformed colony strains^*a*^

Antibiotic	MIC (μg/mL)					
	P4chr1	M	M + OMVs^tet^	M + OMVs^amp^	M + OMVs^chl^	M + OMVs^str^
Tetracycline	32	0.5	0.5	0.5	0.5	1
Ampicillin	128	1	1	1	1	1
Chloramphenicol	32	0.25	0.25	0.25	0.5	0.25
Streptomycin	>512	2	2	2	1	2

a M, Modesto.

*A. paragallinarum* serovars A, B, and C specific antisera were used in the hemagglutination-hemagglutination inhibition (HA-HI) test to serotype the donor strain P4chr1 (serovar A), recipient strain Modesto (serovar C), and ARG-transformed strains. The HI data showed that *A. paragallinarum* Modesto and the transformed colonies had titers similar to those of the serovar C Modesto antiserum and no reaction with the serovar A 0083 and B 0222 antisera.

**FIG 7:**
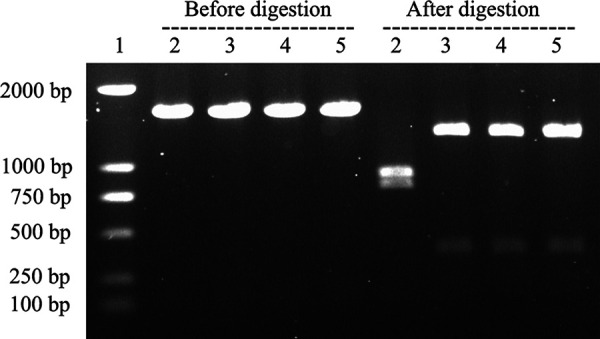
PCR-RFLP profile of the *A. paragallinarum* strains used in this study. Lane 1, marker 2,000 bp; lane 2, P4chr1; lane 3, Modesto; lane 4, Modesto plus OMVs (single colony from TSA plate with antibiotic); lane 5, Modesto plus OMVs (single colony from TSA plate without antibiotic).

These findings were confirmed using the PCR-restriction fragment length polymorphism (RFLP) technique. We found that a 1.6-kb fragment in the hypervariable region of Hmtp210 was amplified for P4chr1, Modesto, and transformed colonies. After digestion with the restriction enzyme Bgl II, the PCR products were divided into two bands, 768 and 868 bp for serovar A and 1,284 and 339 bp in the case of serovar C. We found that the donor bacterium P4chr1 was type A and that both the recipient bacterium Modesto and the transformed colonies were type C (Fig. 7).

## DISCUSSION

Both pathogenic and nonpathogenic Gram-negative bacteria secrete vesicles (11), which contain DNA (plasmid, chromosomal, and/or phage-associated) (11, 20, 21). However, it remains unclear whether OMVs contain a complete genome similar to their parental cells or whether OMVs contain all the genetic information of the bacterial genome. Sequencing purified *A. paragallinarum* OMVs has led to the identification of genomic fragments in OMVs. In this study, we sequenced the multidrug-resistant *A. paragallinarum* strain P4chr1 and its OMVs and performed a comparative genomics analysis between the two genomic sequences (2.69 Mb and 2.77 Mb). We found that the GC content, number of coding genes, and metabolic pathways of the two genomes were very similar. Indeed, magnification of the comparative genomic circle map was required to see the differences. Collinearity analysis revealed that each segment of the OMV genomic sequence could be found in the corresponding region of the P4chr1 genomic sequence. Analysis of whole-genome orthologous clusters has been an important step in comparative genomics research. Identifying clusters between orthologous clusters and constructing networks help explain the functions and evolutionary relationships of proteins across multiple species (22). Here, the whole-genome orthologous gene cluster analysis revealed that the OMVs and P4chr1 had 2,541 homologous gene clusters, with only a few differences, which may be caused by sequencing errors or fragmentation during OMV genome sequencing. Our findings indicated that the genomic sequence of the OMVs was derived from *A. paragallinarum* P4chr1 and that the OMVs had almost complete genomic sequences of *A. paragallinarum* P4chr1, including virulence genes and ARGs. Within the P4chr1 genomic sequence, 11 ARGs were found to be focused in a 25-kb region, forming a structure similar to a tolerance island. BLAST comparison analysis indicated that this sequence was very similar (99%) to a sequence of a field strain of Pasteurella multocida FCf83, isolated from duck in Fujian, China, in December 2015. Furthermore, the gene-coding direction was also the same. These two bacteria have a close genetic affiliation with the same family, *Pasteurellaece*, thereby allowing for easier gene exchange. *A. paragallinarum* and P. multocida are important respiratory pathogens in poultry farms, and they can often be isolated in clinical samples at the same time, which may imply the possibility of horizontal transmission of drug-resistant genes between them (23–25). In addition, the two sequences have the same DNA primase and recombinase before and after, and the same integrase, transposase, recombinase, and endonuclease inside the sequence. Presently, we cannot conclusively determine whether the sequence was transferred by transposition or insertion or possibly through the formation of a cyclized structure from the chromosome.

In recent years, several important functions of OMVs have been reported, including the intra- and interspecies horizontal transfer of ARGs (8). In 2011, Rumbo et al. reported the horizontal transfer of plasmids carrying the carbapenemase resistance gene OXA-24 in OMVs to Acinetobacter baumannii (15). In 2015, Ho et al. demonstrated HGT mediated by Porphyromonas gingivalis OMVs. This bacterium (a *fimA* mutant) carried a 2.1-kb *ermF*-*ermAM* cassette in its *fimA* gene, encoding an erythromycin-resistant gene. The cassette was transferred to the *fimA* gene of another P. gingivalis strain lacking this gene (*erm* gene) via OMVs isolated from the donor strain (18). Finally, in 2019, Fulsundar et al. proposed an optimized and detailed plan to test and confirm that OMV-mediated ARGs can be transferred to Acinetobacter baumannii without plasmids in OMVs (14).

Here, we examined the potential OMV-mediated HGT from the *A. paragallinarum* P4chr1 strain, which contains ARGs to the antibiotic-sensitive *A. paragallinarum* strain Modesto. We found that the antibiotic-sensitive Modesto strain successively survived on antibiotic-treated agar plates with ARGs. Furthermore, its transformed colonies passed several verification tests. ARG-PCR analysis demonstrated that the transformed colonies amplified corresponding ARG products, while the HA-HI and PCR-RFLP assays confirmed that the transformed colonies were derived from the recipient cells and not from donor cells (20, 26). The PCR-RFLP test has some limitations, and our previous studies have discussed the availability of this method (27). In this experiment, PCR-RFLP was suitable for distinguishing serovar A strain P4chr1 from serovar C strain Modesto, and agreed with the result from the HA-HI test. However, the overall ARG transformation efficiency mediated by *A. paragallinarum* OMVs was low compared to that found in previous studies, which reported that transferred ARGs were generally carried in the plasmid (15, 16, 19). Our HGT assays revealed that the highest transformation frequency was 5.42 × 10^−7^ (Table 2) in the streptomycin group and that there was a higher copy number of streptomycin-resistant genes in the donor strain P4chr1. Other studies have suggested that OMVs contain only partial genomic fragments, such that some fragments do not contain intact ARGs, and not every vesicle contains DNA (28). Tran and Boedicker hypothesized that the ability to acquire DNA may depend on the species of the donor/recipient bacteria (29).

In our antimicrobial susceptibility test, the MIC values produced by the transformed colony strains did not increase compared with the antibiotic-susceptible strain Modesto, suggesting that the ARGs transferred by donor OMVs are not persistent in recipient cells after passaging. This unexpected result exposed a poor passaging ability of the transformed colonies. It has been proposed that once a gene has been transferred into its recipient, it must be integrated into the chromosomal DNA in order to persist within the cells (18). Ho et al., for example, demonstrated that the *erm* gene in the vesicles of the *fimA* mutant was flanked with *fimA* sequences at both ends and that homologous DNA recombination occurred between the vesicle donor DNA and the chromosomal DNA of the recipient (18).

Since no homologous sequences were flanked with drug-resistant genes in the recipient bacterium Modesto (which was confirmed by our genomic sequencing data) in our study, the ARGs could not have been incorporated into the chromosomes of recipient cells through homologous recombination (18). Moreover, even if homologous sequences were present, the likelihood of gene recombination between vesicle donor DNA and recipient chromosomal DNA is low and not as effective as the transfer of genes by plasmids, which has been demonstrated in multiple HGT studies (15, 16).

**Conclusion.** In this study, we present the complete genome sequencing data of *A. paragallinarum* P4chr1 and its OMVs and confirm that they are highly homologous. In addition, we identified some drug resistance genes in the *A. paragallinarum* P4chr1 genome that were not present in the *A. paragallinarum* Modesto genome. Using purified OMVs from P4chr1 as the vector, four AGRs were transferred into the drug-sensitive *A. paragallinarum* Modesto strain. However, the ARG transformation efficiency and persistency were limited. More studies are required to further understand OMV-mediated HGT with chromosomal DNA-based ARGs.

## MATERIALS AND METHODS

### Bacterial strains.

*A. paragallinarum* P4chr1 was isolated from the infraorbital sinus sample of a diseased bird from a chicken farm in China in 2021. It was identified as serovar A earlier with a conventional hemagglutination-hemagglutination inhibition (HA-HI) test (20). *A. paragallinarum* serovar-specific antisera against reference strains 0083 (antiserum A), 0222 (antiserum B), and Modesto (antiserum C) were prepared previously in this laboratory. 16S rRNA gene sequencing and biochemical analyses were used to identify the bacterial species. *A. paragallinarum* Modesto is a serovar C reference strain preserved in the laboratory (GenBank accession number CP086713).

### Isolation and purification of OMVs.

The isolation and purification steps for OMVs were modified from a previously published procedure (30). Briefly, *A. paragallinarum* strain P4chr1 was inoculated in tryptic soy broth (TSB) containing 10% (vol/vol) chicken serum and 0.0025% (wt/vol) NAD and cultured for 15 h at 37°C and 180 rpm. The cultured liquid was centrifuged for 30 min at 7,500 × *g* at 4°C, and the supernatant was filtered through a Stericup filter (Millipore Corporation, Massachusetts, USA) with a pore diameter of 0.45 μm to remove bacteria suspended in the broth. The filtered supernatant was centrifuged for 3 h at 150,000 × *g* at 4°C (SW40 Ti rotor; Beckman-Coulter, Germany). Then, the supernatant was discarded, and the precipitate was resuspended in 30 mL 0.05 mol/L Tris-HCl buffer (pH 8.0). This process was repeated, and the pellet was resuspended in 5 mL phosphate-buffered saline (PBS) to obtain crude OMVs. The extracted OMVs were centrifuged for 1 h at 75,000 × *g* at 4°C. Finally, purified OMVs were obtained by resuspending the collected pellet in 2 mL 50 mM HEPES-150 mM NaCl solution.

### DNA preparation and sequencing of OMVs.

DNA was extracted as previously described (20) and sequenced via second-generation sequencing methods (Allwegene Technologies, China).

### Transmission electron microscopy.

The vesicle suspension was fixed in cold 2.5% (vol/vol) glutaraldehyde for 2 h at 4°C, followed by 1% (wt/vol) osmium tetroxide for 1 h at 4°C. After washing with deionized water, the immobilized vesicles were placed on a 200-mesh grid and imaged using a Philips CM 100 transmission electron microscope (TEM) at 80 kV.

### Whole-genome sequencing (WGS) and bioinformatic analysis of *A. paragallinarum* P4chr1.

Bacterial genomic DNA was extracted using the Invitrogen DNA minikit (Thermo Fisher Scientific, USA). *A. paragallinarum* P4chr1 was subjected to WGS using a combination of Nanopore PromethION (Oxford Nanopore Technologies, Beijing, China) and Illumina NovaSeq 6000 (Genewiz, Beijing, China) platforms. Canu v1.5 and Falcon v0.3.0 were used to perform mixed assembly of the original data. The second-generation sequencing-derived small fragment data were used to perform single-base correction (GATK) on the assembly to obtain a high-confidence assembly sequence. Gene prediction was performed using Prodigal software (PROkaryotic DYnamic programming Gene-finding ALgorithm), since Prodigal has high-quality gene structure prediction and better translation initiation site prediction, and gives fewer false positives than other software (31). Prodigal, Glimmer, and GeneMark.hmm were used for gene prediction of NCBI (National Center for Biotechnology Information) prokaryotes. Genomes were annotated using the online database RAST (http://rast.nmpdr.org/), and the results were corrected using the BLASTn database (https://blast.ncbi.nlm.nih.gov/Blast.cgi). The ResFinder database was used to detect ARGs in the genome (https://cge.cbs.dtu.dk/services/). Gene function and metabolic pathway predictions were obtained using the Blast2GO annotation pipeline. The BRIG (BLAST Ring Image Generator) tool was used to draw the circular map of *A. paragallinarum* P4chr1 and compare it with the OMVs. Transmembrane domains (TMDs) in the P4chr1 genome were predicted using TMHMM Server v.2.0. Finally, the software NCBI BLAST+ was used to compare amino acid sequences of the proteins with the data from the COG, KEGG, VFDB, Nt, Nr, and Swiss-Prot databases to obtain the protein function annotation information.

### Recipient cell preparation.

*A. paragallinarum* Modesto was grown in 10 mL TSB with the supplements described earlier for 15 h at 37°C and 180 rpm. The 2% (vol/vol) culture was then transferred to 500 mL TSB containing the same supplements and incubated to an optical density (OD) of 0.4 to 0.6. Next, the culture was centrifuged for 30 min at 4,000 × *g* at 4°C, and the supernatant was discarded. The precipitate was resuspended in 10 mL precooled 272 mM sucrose solution. The culture was centrifuged for 30 min at 4,000 × *g*, and then the precipitate was resuspended in 1 mL cooled 10% (vol/vol) glycerol and divided into 200-μL aliquots.

### OMV-mediated gene transfer.

**FIG 8:**
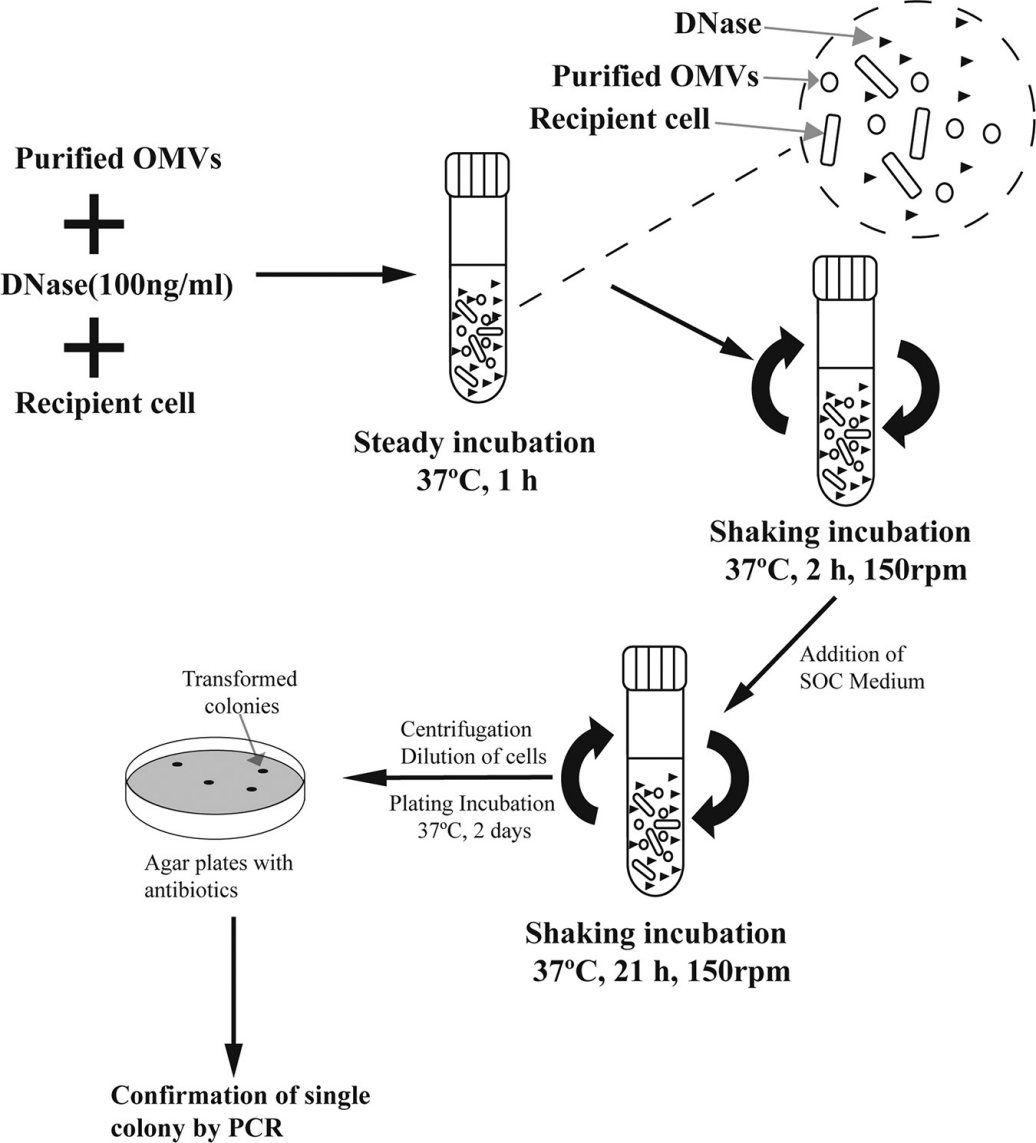
Schematic presentation of the steps involved in vesicle-mediated transfer in an *A. paragallinarum* strain (12).

The transformation experiment was performed as described previously by Fulsundar et al. (14) (Fig. 8). Experiments were conducted in triplicate three independent times. To prepare the gene transfer incubation mixture, 50 μL of recipient Modesto cells were added to 500 μL super optimal broth with catabolite repression (SOC; 10% [vol/vol] chicken serum and 0.0025% [wt/vol] NAD) medium supplemented with chicken serum and NAD in each Eppendorf tube, followed by addition of 500 μL purified OMVs with known protein concentration (measured with a Bradford assay kit and adjusted to 1 mg/mL). Next, 1 μL 100 μg/μL DNase (final concentration 100 ng/mL) (Thermo Fisher Scientific, California, USA) was added. The tubes were statically incubated for 1 h at 37°C, and then the mixture was transferred aseptically to culture tubes and incubated for a further 2 h with shaking at 150 rpm. Then, 2 mL SOC medium was added, and samples were incubated with shaking for an additional 21 h. Next, the cells were pelleted by centrifugation, resuspended in 1 mL SOC medium, and plated on TSA plates with or without antibiotics. The four test group cells were plated on TSA with four different antibiotics: chloramphenicol, erythromycin, tetracycline, or streptomycin. Three control groups were also prepared. Control A was used to determine the number of receptor cells (CFU/0.1 mL). Control A cells were prepared in the same way as the test groups but did not contain DNase in their sample mixture and were 10 times serial diluted for viable counting before being plated on TSA plates in the absence of antibiotics. Control B was used to assess the effect of DNase on recipient cells. Control B cells were prepared in the same way as the test groups but were plated on antibiotic-free TSA plates. Control C was the negative control. Control C contained only recipient cells and was plated on TSA plates containing the four antibiotics used in the test groups. The plates were incubated for 2 days at 37°C and then evaluated by counting the number of colonies or transformed colonies grown on each plate for every group. The transformation frequency was calculated as the number of transformed colonies over the number of recipient cells.

### Confirmation of gene transfer by PCR.

Based on the P4chr1 genomic information and NCBI ARG sequence (WP_001089068, WP_032491311, WP_000214125, and WP_010890156), four pairs of ARG primers were designed and synthesized (Table 4). The PCR system was 50 μL. The amplification steps were as follows: 95°C for 5 min; 30 cycles of 95°C for 30 s, 55°C for 30 s, and 72°C for 1 min; and a final step at 72°C for 10 min. PCR products were analyzed by agarose gel electrophoresis. The PCR products were purified and sent to a company for cloning and sequencing (Sangon, Shanghai, China). The sequence information was acquired by aligning the results with sequences obtained from GenBank using BLAST (www.ncbi.nlm.nih.gov/BLAST/). The donor bacterium P4chr1 containing DNA with ARGs was used as the positive control, and water was used as the negative control.

**TABLE 4:** Primer sets used for amplification of the DNA fragment

No.	Gene	Primer	Sequence	Product (bp)	Reference or source
1	*tetB*	tetb-F	TAGGGGTTGAGACGCAATCG	372	This study
		tetb-R	CAGGTAAAGCGATCCCACCA		
2	*bl2d_oxa1*	bl2d_oxa1-F	GCAAAGTGTGCAACGCAAAT	485	This study
		bl2d_oxa1-R	GCTGTGAATCCTGCACCAGT		
3	*cml_e3*	cml_e3-F	GCCTTTGTTGCGTTTCGTCT	516	This study
		cml_e3-R	CCATCTGGCGACAAAGGACT		
4	*aph33ib*	aph33ib-F	AATGCCGTCAATCCCGACTT	386	This study
		aph33ib-R	CAACCCCAAGTCAGAGGGTC		
5	*Hmtp210*	210-F	GATGGCACAATTACATTTACA	1,600	26
		210-R	ACCTTGAGTGCTAGATGCTGTAGGTGC		

### Confirmation of recipient cell by serotyping and PCR-RFLP.

The classical hemagglutination-hemagglutination inhibition (HA-HI) test was conducted as described previously (20). HA antigens were prepared from TSB cultures seeded with acquired transformed colonies, donor strain P4chr1 (serovar A), and recipient strain Modesto (serovar C). PCR-restriction fragment length polymorphism (RFLP) analysis (26) was performed on the recipient bacterium, donor bacterium, and colonies on the resistant plates as described above.

### Antimicrobial susceptibility testing.

*A. paragallinarum* P4chr1, Modesto, and transformed colonies were cultured in TSB containing supplements, in the absence of antibiotics. Antimicrobial susceptibility testing was performed using a broth microdilution method according to the protocol described by the Clinical and Laboratory Standards Institute (CLSI) (32). The resultant MIC data were interpreted according to the recommendations outlined in CLSI documents VET08 (32) and M100 (33). E. coli ATCC 29213 served as the quality control strain.

### Data availability.

The complete genomic sequence of the chromosomal DNA P4chr1 has been deposited in GenBank under the accession number CP081939. The genomic sequence of its OMVs has been deposited under BioSample number SAMN22170838.

We confirm that the data supporting the findings of this study are available within the article, its supplemental materials, and NCBI (GenBank accession number CP081939, BioSample accession number SAMN22170838).
